# Clinical and genetic analysis of Chinese patients with Leigh syndrome caused by biallelic loss-of-function variants of the *NDUFAF6* gene

**DOI:** 10.3389/fneur.2026.1778719

**Published:** 2026-03-30

**Authors:** Qi Yang, Qiang Zhang, Xunzhao Zhou, Zailong Qin, Xuanjing Liang, Sheng Yi, Shujie Zhang, Weiliang Lu, Shang Yi, Jingsi Luo

**Affiliations:** 1 Guangxi Key Laboratory of Birth Defects Research and Prevention, Guangxi Key Laboratory of Reproductive Health and Birth Defects Prevention, Maternal and Child Health Hospital of Guangxi Zhuang Autonomous Region, Nanning, China; 2 Department of Genetic and Metabolic Central Laboratory, Maternal and Child Health Hospital of Guangxi Zhuang Autonomous Region, Nanning, China; 3 Department of Radiology, Maternal and Child Health Hospital of Guangxi Zhuang Autonomous Region, Nanning, China; 4 Guangxi Clinical Research Center for Pediatric Diseases, Maternal and Child Health Hospital of Guangxi Zhuang Autonomous Region, Nanning, China

**Keywords:** complex I deficiency, Leigh syndrome, *NDUFAF6* gene, novel variant, whole-exome sequencing

## Abstract

Leigh syndrome (LS) is the most common pediatric mitochondrial disorder, typically presenting in infancy with developmental regression, neurological dysfunction, and characteristic brain MRI lesions. It is linked to over 110 genes affecting cellular energy production, making it highly genetically heterogeneous, with complex I deficiency being the most frequent cause. Biallelic mutations in *NDUFAF6*—a key assembly factor of complex I—cause autosomal recessive Leigh syndrome, specifically NDUFAF6-related Leigh syndrome, also designated as mitochondrial complex I deficiency, nuclear type 17 (MC1DN17; OMIM 618239). Herein, we describe two patients with biallelic loss-of-function variants in *NDUFAF6*. Patient 1 was homozygous for an in-frame duplication (c.362_364dupTGG; p. Val121dup), whereas patient 2 carried this duplication in trans with a novel frameshift variant (c.169_190dup; p. Leu64fs*2). Both patients manifested motor deterioration, dystonia, dysphagia, and elevated blood lactate levels during infancy, along with symmetrical basal ganglia necrosis on brain MRI. A retrospective analysis of all 24 MC1DN17 cases confirmed infantile/childhood onset, psychomotor regression, dystonia, bilateral striatal necrosis with additional features, and hyperlactataemia as universal characteristics. Mortality was low (1/24; 4%), with motor function maintained for longer than in some other LS-associated genetic subtypes. No clear genotype–phenotype correlation was identified, and disease progression remains difficult to predict. There are currently no disease-modifying treatments available; only supportive care can be provided. Our study expands the *NDUFAF6* mutational spectrum and consolidates its distinct clinical profile, highlighting the need for long-term data to define natural history and guide therapy.

## Introduction

Leigh syndrome (LS, OMIM #256000) is a severe, early-onset progressive neurodegenerative disorder and the most common mitochondrial disease phenotype in pediatric populations, with an estimated prevalence of 1 in 40,000 live births (1). It is clinically characterized by psychomotor delay or regression, hypotonia, dystonia, ataxia, spasticity, seizures, and brainstem dysfunction, often presenting in infancy or childhood and following an episodic or stepwise deteriorating course, frequently triggered by metabolic stress such as infections (2, 3). Neuropathologically, LS is defined by bilateral, symmetrical necrotic lesions in deep gray matter structures, including the basal ganglia, brainstem, thalamus, and spinal cord, typically observed via cranial magnetic resonance imaging (4). The diagnostic approach combines these characteristic clinical and neuroradiological findings with identification of a pathogenic genetic variant and often includes biochemical support such as elevated lactate in blood or cerebrospinal fluid (CSF) and/or evidence of defective oxidative phosphorylation (OXPHOS) (5).

LS is exceptionally genetically heterogeneous, with over 110 known disease-associated genes encoded by both nuclear (nDNA) and mitochondrial (mtDNA) genomes (4, 6, 7). These gene mutations disrupt proteins that are either structural subunits of the mitochondrial respiratory complexes or critical assembly factors, ultimately leading to failure of the OXPHOS system and impaired cellular ATP production. A defect in Nicotinamide adenine dinucleotide (NADH)-ubiquinone oxidoreductase or NADH dehydrogenase (complex I) is the most common biochemical cause, accounting for 35–50% of LS cases (8, 9).

Complex I, the largest enzyme complex of the respiratory chain, contains 44 subunits and requires numerous assembly factors for its biogenesis (10–12). Of these, the nuclear-encoded protein NADH dehydrogenase (ubiquinone) complex I assembly factor 6 (NDUFAF6, also known as C8orf38) plays a vital and highly conserved role in the initial stages of complex I assembly (13–16). This is particularly important for the biogenesis and stabilization of the mitochondrial DNA-encoded MT-ND1 subunit. Biallelic mutations in *NDUFAF6* are a recognized cause of Leigh syndrome due to complex I deficiency (13, 14, 17, 18). NDUFAF6-related Leigh syndrome, also known as Mitochondrial complex I deficiency, nuclear type 17 (MC1DN17, OMIM 618239) is classically characterized by early-onset neurodevelopmental regression, MRI evidence of brainstem and basal ganglia dysfunction, lactic acidosis and elevated lactate levels in the plasma and/or cerebrospinal fluid. Currently, most cases remain without effective therapeutic interventions, and the disease tends to progress rapidly and have an unfavourable prognosis. Therefore, detailed genetic and clinical descriptions of additional cases are crucial for enhancing the diagnostic efficiency, genetic counselling, and prenatal screening standards for this condition. Herein, we report two additional cases of NDUFAF6-related Leigh syndrome in Chinese patients. Molecular genetic testing revealed a novel *NDUFAF6* variant. By combining previously reported cases, we also conducted a systematic analysis of their clinical, biochemical, and genetic characteristics, thereby deepening the understanding of the disease.

## Materials and methods

### Ethical compliance

This study received approval from the Institutional Review Board and Ethics Committee of Guangxi Maternal and Child Health Hospital. Written informed consent for genetic testing was provided by the parents or guardians of all children.

### Search strategy

we conducted systematic searches in PubMed, Web of Science, Scopus, and Google Scholar from inception through December 2025 using combinations of terms including “*NDUFAF6*,” “MC1DN17,” “mutation,” “mitochondrial complex I deficiency,” and “Leigh syndrome,” supplemented by manual screening of reference lists. Studies were included if they reported individual patients with confirmed biallelic pathogenic variants in *NDUFAF6* and provided clinical, genetic, and/or functional data, while we excluded cases without genetic confirmation, duplicate reports of the same patient, review articles, conference abstracts, and non-English publications. Two reviewers independently assessed eligibility with consensus resolution.

### Whole exome sequencing and sanger sequencing

**Figure 1:**
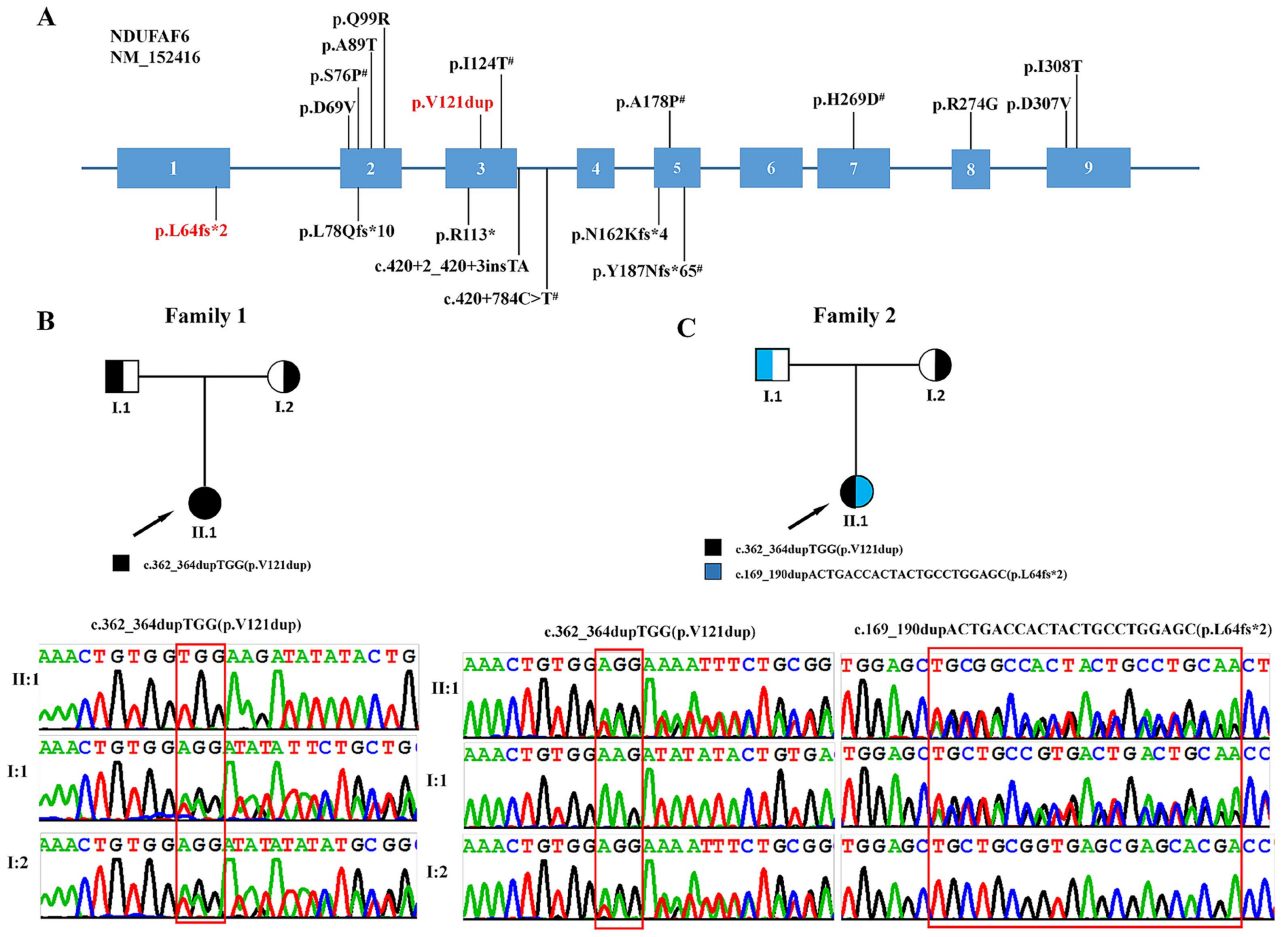
*NDUFAF6* variant features. **(A)** Distribution of all *NDUFAF6* pathogenic variants. All 18 reported variants are shown (red = the variants identified in this study; # = recurrent variants, allele count>2). **(B,C)** Pedigree of affected individuals. Squares indicate males and a circle for females. Arrows indicated for proband. Variants were identified by the Sanger sequencing.

Genomic DNA was isolated from 2 mL of peripheral blood lymphocytes collected from patients and theirs family members using the Lab-Aid DNA kit (Zeshan Biotechnology Co., Ltd., Xiamen, China) following the manufacturer’s instructions (Figure 1). Trio-based whole-exome sequencing (trio-WES) was performed using an Agilent SureSelect V5 enrichment capture kit (Agilent Technologies, Santa Clara, CA, USA), followed by 100 bp paired-end sequencing on the Illumina HiSeq 2000 platform (Illumina, San Diego, CA, USA). Sequencing reads were aligned to the hg19/GRCh38 human reference genome using the Genome Analysis Toolkit (GATK, version 3.4; Broad Institute, Cambridge, MA, USA). Variant calling and annotation were conducted with TGex software, which prioritized variants with a minor allele frequency (MAF) of ≤0.001 in public databases such as the 1,000 Genomes Project, Exome Sequencing Project (ESP), and Exome Aggregation Consortium (ExAC). The functional implications of candidate variants were evaluated using a suite of in silico prediction tools, namely REVEL, Polyphen2, SIFT, LRT, Variant Assessor, CADD, and MutationTaster. Furthermore, a three-dimensional model of the NDUFAF6 protein was generated with SWISSMODEL.^1^ Finally, the pathogenicity of these variants was interpreted based on the standards and guidelines established by the American College of Medical Genetics and Genomics and the ClinGen Sequence Variant Interpretation Working Group (19).

## Results

**Figure 2:**
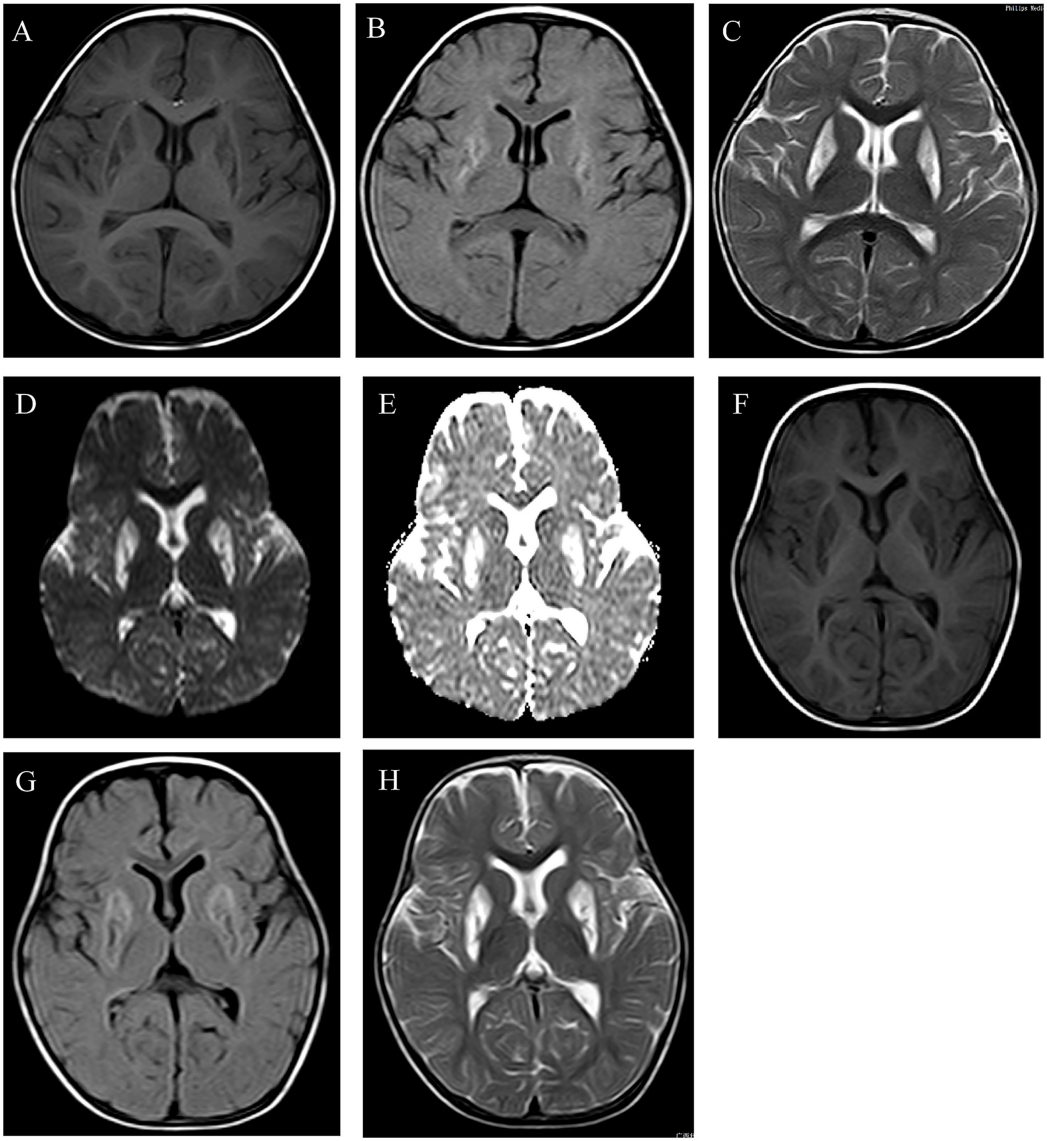
Brain imaging manifestations of patients. MRI images of the patient 1 at 15 months of age **(A–C)**. Symmetrical patchy and small patchy areas of prolonged T1 and T2 signal intensity are observed in both putamen and thalamus. MRI images of the patient 1 at 15 months of age **(D–H)**. Multiple symmetrical abnormal signal lesions are visible in both frontal lobes and basal ganglia regions. These lesions exhibit predominantly high signal intensity on diffusion-weighted imaging (DWI) **(D)**, and low signal intensity on the diffusion coefficient (ADC) map **(E)**, low signal intensity on T1-weighted images (T1WI) **(F)**, predominantly high signal intensity on FLAIR sequences with a minority showing low signal intensity **(G)**, and high signal intensity on T2-weighted images (T2WI) **(H)**.

Patient 1 was first admitted to the Department of Paediatric Rehabilitation, Guangxi Zhuang Autonomous Region Maternal and Child Health Hospital, for developmental regression (HP:0002376) (Figure 1A). She was a 15-month-old girl who is the first child of non-consanguineous parents. She was born full-term by vaginal delivery with a birth weight of 3.15 kg. There were no perinatal complications and no significant family history. Developmental milestones were normal prior to the onset of symptoms, lifting the head at 3 months, saying “dad” and “mom” at 10 months, and standing with support at 11 months. The initial symptoms emerged at approximately 1 year of age, manifested as an inability to crawl or stand with support (HP:0030174), hand tremors (HP:0002378) when reaching for objects, along with significant orobulbar involvement resulting in speech impairments including dysarthria (HP:0001260) and stuttering (HP:0025265), as well as dysphagia (HP:0002015). These symptoms progressed gradually over time. She also exhibited limb dystonia (HP:0001332), with hypotonia (HP:0001252) at rest and marked hypertonia (HP:0001276) during stress or attempted movement. Biochemical tests revealed an elevated serum lactate (HP:0002151) level of 3.8 mmol/L (normal < 2.0 mmol/L), suggesting metabolic acidosis (HP:0001942), while all other metabolic parameters remained within normal range. When she was 15 months old, her development was assessed using the Gesell Developmental Diagnostic Scale. Her domain scores were as follows: gross motor skills (29), fine motor skills (37), adaptive skills (32), language skills (50), and personal-social skills (42). Brain MRI at 15 months of age revealed symmetrical abnormal signals in the bilateral putamen and thalamus (HP:0012759) (Figures 2A–C).

Patient 2 was referred to the Department of Paediatric Neurology at Guangxi Zhuang Autonomous Region Maternal and Child Health Hospital at 1 year and 3 months of age due to developmental regression (HP:0002376) (Figure 1C). She was the first child of non-consanguineous parents, born at 39 weeks of gestation by full-term spontaneous delivery, with birth weight and length both within normal ranges. Before the age of 12 months, she had demonstrated normal motor, cognitive, social and language development. However, regression then began, manifesting as unsteady sitting (HP:0031936), an inability to stand (HP:0030174), reduced finger dexterity (HP:0007010), a loss of vocabulary (HP:0002371) except for monosyllables, and an impaired comprehension of simple commands (HP:0002376). After diagnosis she began daily physical therapy, occupational training, and an oral nutritional supplement (biotin 10 mg, thiamine 500 mg, coenzyme Q10 100 mg, levocarnitine 100 mg with breakfast). Despite uninterrupted therapy her motor function continued to deteriorate (HP:0002333). At 16 months of age, her development was assessed using the Gesell Developmental Diagnostic Scale, which yielded a Developmental Quotient (DQ; scores <70 indicate delay). The domain scores were as follows: gross motor (26), fine motor (39), adaptive (35), language (48), and personal-social (39). Metabolic analysis results were normal, with only serum lactate elevated (HP:0002151) to 6.48 mmol/L (normal range <2.0 mmol/L), consistent with metabolic acidosis (HP:0001942). Brain MRI at 16 months of age revealed symmetrical abnormal signals in the bilateral frontal lobes and basal ganglia regions (HP:0012759) (Figures 2D–H).

### Molecular analysis

**Figure 3:**
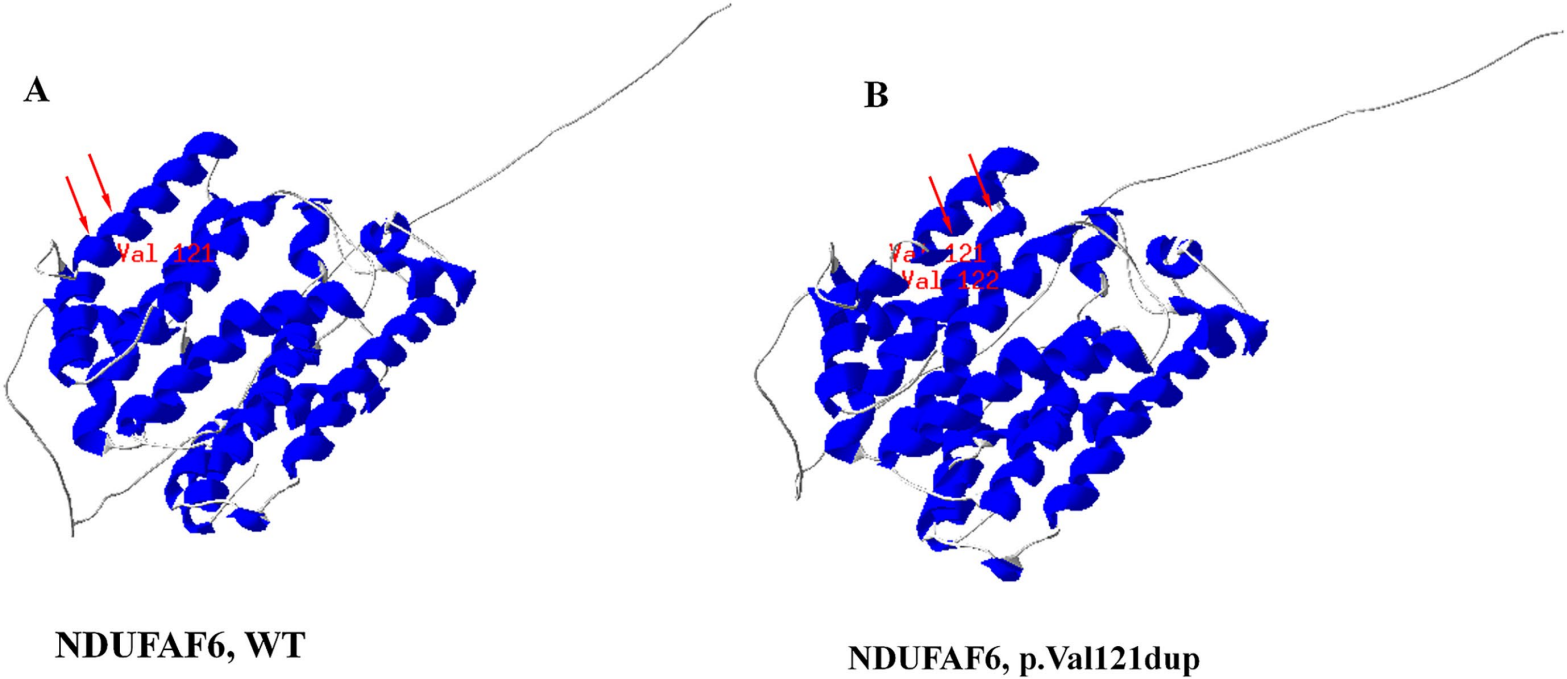
Three-dimensional structures of NDUFAF6 protein. **(A)** Wild-type, **(B)** c.362_364dupTGG (p. Val121dup), mutant-type. The arrows indicated the location of p. Val121 and p. Val122.

Whole-exome sequencing (WES) was performed on both probands to identify pathogenic variants causing the disease. Sequencing generated approximately 6.5 Gb of read data for each sample, with mean target region coverage exceeding 98% and over 97% of bases covered at a minimum depth of 20×. A total of 25,061 and 24,360 coding region or splice site (±10 bp) single nucleotide variants (SNVs) and insertions/deletions (indels) were identified in Patient 1 and Patient 2, respectively. By filtering for rare variants [minor allele frequency (MAF) > 1% in gnomAD, dbSNP132, the Exome Sequencing Project (ESP), the 1,000 Genomes Project, and an in-house database], the number was reduced to 725 and 734 unique SNVs. After excluding synonymous variants and computationally predicted benign missense variants, 416 and 425 candidate variants remained. Phenotype-driven analysis using the TGex platform^2^ was subsequently performed. For Patient 1, four candidate genes were identified: *NDUFAF6*, *SCO2*, *DNA2*, *CDK5RAP2*, and *ZAP70*. Heterozygous variants in *CDK5RAP2* and *ZAP70* were excluded because their associated diseases follow an autosomal recessive inheritance pattern. Variants in *SCO2* and *DNA2* were inherited from the unaffected father and were therefore excluded. For Patient 2, five candidate genes were identified: *NDUFAF6*, *SET*, *COL18A1*, *FDFT1*, and *KMT2D*. The heterozygous variant in *FDFT1* was excluded because its associated disease follows an autosomal recessive inheritance pattern. Variants in *SET* and *COL18A1* were inherited from the unaffected father, and the variant in *KMT2D* was inherited from the unaffected mother, and were therefore excluded. Then, whole-exome sequencing identified two novel variants in the *NDUFAF6* gene (NM_152416.4) in two patients. Patient 1 was homozygous for c.362_364dupTGG (p. Val121dup), while patient 2 was compound heterozygous for c.362_364dupTGG (p. Val121dup) and c.169_190dupACTGACCACTACTGCCTGGAGC (p. Leu64fs*2) (Figures 1B,C). Both variants were validated by Sanger sequencing, and their parental origin was confirmed by sequencing the parents’ samples. The parents of Patient 1 were both heterozygous carriers of the c.362_364dupTGG (p. Val121dup) variant. The father of Patient 2 was heterozygous for the c.169_190dupACTGACCACTACTGCCTGGAGC (p. Leu64fs2) variant, and the mother was heterozygous for the c.362_364dupTGG (p. Val121dup) variant. In silico prediction tools classified both as deleterious. According to the ACMG/AMP standards and guidelines, c.362_364dupTGG (p. Val121dup) was classified as ‘likely pathogenic’ based on PM2, PM3, PM4 and PP4 criteria (Table 1). c.169_190dupACTGACCACTACTGCCTGGAGC (p. Leu64fs*2) was classified as ‘pathogenic’ based on PVS1, PM2 and PP4 criteria (Table 1). To further analyze the impact of the amino acid change caused by the c.362_364dupTGG (p. Val121dup) variant on protein structure, we modeled it into the wild-type NDUFAF6 crystal structure. The p. Val121 residue forms part of a multi-helical structure (Figure 3A); however, p. Val121dup leads to a structural defect in this region (Figure 3B).

**Table 1:** Predicted pathogenicity of de novo *NDUFAF6* variant.

Gene	Variant (NM_145331.2)	LRT	Mutationtaster	NMDEscPredictor	ACMG/AMP
*NDUFAF6*	c.362_364dupTGG (p. Val121dup)	NA	D	NA	LP(PM2 + PM3 + PM4 + PP4)
*NDUFAF6*	c.169_190dupACTGACCACTACTGCCTGGAGC (p. Leu64fs*2)	D	D	NMD	P(PVS1 + PM2 + PP4)

D, deleterious or damaging; NA, not available; NMD, Nonsense Mediated Decay; LP, Likely pathogenic; P, pathogenic.

## Discussion

NDUFAF6-related Leigh syndrome, also known as Mitochondrial complex I deficiency, nuclear type 17 (MC1DN17, OMIM 618239), is a rare autosomal recessive disorder of mitochondrial energy metabolism. It results from pathogenic biallelic variants in *NDUFAF6*, an assembly factor essential for the biogenesis of respiratory-chain complex I (NADH:ubiquinone oxidoreductase) (13, 14, 17, 18). Clinically, patients present with the characteristic neuroradiological findings of Leigh syndrome in infancy or early childhood, including progressive psychomotor regression, brainstem and basal ganglia dysfunction evident on magnetic resonance imaging, lactic acidosis with elevated lactate in plasma and cerebrospinal fluid. Additional features often include dystonia, dysphagia, and intermittent metabolic crises triggered by concurrent infections. To date, only 22 cases have been reported, and further investigation is required into its natural course, genotype–phenotype associations, and optimal management strategies (Table 2) (17, 18, 20–28). In this study, two unrelated Chinese patients were diagnosed with MC1DN17 after whole-exome sequencing (WES) revealed two *NDUFAF6* gene variants.

**Table 2:** Clinical, biochemical and radiological features of patients with *NDUFAF6* variants reported in the literature.

Patients clinical data	This study		Pagliarini et al. (18)		Martikainen et al. (20)		Bianciardi et al. (21)	Kohda et al. (17)				Fang et al. (22)	Catania et al. (23)		
	P 1	P 2	P 3	P 4	P 5	P 6	P 7	P 8	P 9	P 10	P 11	P 12	P 13	P 14	P 15
Variants in NDUFAF6 (NM_152416.4)	c.362_364dupTGG(p. V121dup)	c.169_190dupACTGACCACTACTGCCTGGAGC (p. L64fs*2);c.362_364dupTGG(p. V121dup)	c.296A > G(p. Q99R)	c.296A > G(p. Q99R)	c.226 T > C(p. S76P)	c.226 T > C(p. S76P)	c.532G > C(p. A178P)	c.371 T > C(p. I124T); c.805C > G(p. H269D)	c.820A > G(p. R274G)	c.226 T > C(p. S76P); c.805C > G(p. H269D)	c.206A > T(p. D69V); c.371 T > C(p. I124T)	c.337C > T(p. R113*) ; c.265G > A(p. A89T)	c.532G > C(p. A178P);c.420 + 784C > T	c.532G > C(p. A178P);c.420 + 784C > T	c.532G > C(p. A178P);c.420 + 784C > T
Age at onset (m)	12	12	10	7	12	12	42	72	17	0	2	48	21	12	60
Age of dead	alive	alive	34 months	alive	alive	alive	alive	NA	NA	NA	NA	NA	alive	alive	alive
Age at last examination	1y 3 m	1y 4 m	−	1 y 10 m	4 y	NA	8 y 6 m	NA	NA	NA	NA	NA	5 y	3 y	11 y
Clinical features Onset	Insidious	Insidious	NA	NA	NA	NA	Insidious	NA	NA	NA	NA	NA	Acute/ febrile illness	Insidious	Insidious
Neurological regression	+	+	+	+	+	+	+	+	+	+	+	NA	+	+	+
Extrapyramidal features	Dystonia, Unable to crawl or stand with support; hands tremble when reaching for objects	Unsteady sitting, difficulty standing, poor finger dexterity,	Decreased movement and strength and rigidity	Decreased movement and strength and rigidity	Dystonia	Dystonia	Dystonia	NA	NA	NA	NA	NA	Tremor, hypertonia	+	Oculomandibular dystonia, extrapyramidal hypertonia and a mild camptocormic gait
Cerebellar ataxia	+	N	+	+	NA	NA	−	NA	NA	NA	NA	NA	+	+	−
Dysarthria	+	N	NA	NA	NA	NA	+	NA	NA	NA	NA	NA	NA	−	+
Basal ganglia necrosis	Caudate and putamen	Frontal lobes and basal ganglia regions	Putamen	Caudate and putamen	Caudate and putamen	Caudate and putamen	Caudate and putamen	NA	+	NA	+	NA	Caudate and putamen	+	Putamen
Subcortical white matter	−	−	Parietal white matter	Normal	+	+	Normal	NA	NA	NA	NA	NA	NA	NA	−
Spinal cord	NA	NA	Dorsal pons	−	NA	NA	−	NA	NA	NA	NA	NA	NA	NA	NA
Cerebelar involvement	NA	NA	NA	NA	NA	NA	Dentate nuclei	NA	NA	NA	NA	NA	T2- hyperintensities in dentate nuclei and superior cerebellar peduncles.	T2- hyperintensities in dentate nuclei and superior cerebellar peduncles.	−
MR spectroscopy lactate peak	NA	NA	NA	NA	NA	NA	+	NA	NA	NA	NA	NA	NA	NA	NA
Biochemical investigation Lactate in plasma	3.8 mmol/L	6.48 mmol/L	Elevated	Elevated	NA	NA	Elevated	NA	NA	Elevated	NA	1.4–2.08 - mmol/L	2.8 mmol/ L	2.2 mmol/L	Normal
MRC CI (% of residual activity):	NA	NA	36% in muscle 14% in fibroblasts 20% in liver	14% in fibroblasts	NA	NA	72% in muscle 18% y 32% in fibroblasts*	(< 30% in a tissue, < 40% in a fibroblast cell line, or < 40% in _2 tissues)	< 20% in a tissue, < 30% in a fibroblast cell line, or < 30% in _2 tissues)	(< 20% in a tissue, < 30% in a fibroblast cell line, or < 30% in _2 tissues)	(< 20% in a tissue, < 30% in a fibroblast cell line, or < 30% in _2 tissues)	NA	42% in muscle 38% in fibroblasts	NA	−
Other symptoms	−	−	Seizures	Seizures	NA	NA	Decreased fine manual motor abilities, scoliosis	−	Muscle atrophy	−	−	Excercise intolerance, weakness	Drooling	−	Drooling

Patients clinical data	Baide-Mairena et al. (24)			Tian et al. (25)		Johnstone et al. (26))	Kim et al. (27)	Zhou et al. (28)		Total
	P 16	P 17	P 18	P 19	P 20	P 21	P 22	P 23	P 24	*p* = 24
Variants in CCDC47 (NM_020198)	c.371 T > C(p. I124T),/ c.554_558delTTCTT(p. Y187Nfs*65)	c.371 T > C(p. I124T),/ c.554_558delTTCTT(p. Y187Nfs*65)	c.371 T > C(p. I124T); c.554_558delTTCTT(p. Y187Nfs*65)	c.371 T > C(p. I124T), c.485_486insA(N162Kfs*4	c.371 T > C(p. I124T), c.485_486insA(N162Kfs*4	c.371 T > C(p. I124T), c.420 + 2_420 + 3insTA	c.371 T > C(p. I124T); c. c.233_242del(p. L78Qfs*10)	c.371 T > C(p. I124T); c.923 T > C(p. I308T)	c.371 T > C(p. I124T); c.920A > T(p. D307V)	Missense = 10, Frameshift = 4, Splicing = 2, Small in-frame duplication = 1
Age at onset (m)	30	30	17	48	24	48	36	43	24	
Age of dead	alive	alive	alive	alive	alive	alive	alive	alive	alive	dead = 1, alive = 18
Age at last examination	7 y	6 y 6 m	6 y 6 m	10 y	6 y	2 y 3 m	4 y 8 m	6 y 4 m	2 y	
Clinical features Onset	Insidious	Insidious	Insidious	Acute/ febrile illness	Acute/ febrile illness	Acute/ febrile illness	Insidious	Insidious	Insidious	Insidious = 11, Acute/ febrile illness = 4
Neurological regression	+	+	+	+	+	+	+	+	+	23/23
Extrapyramidal features	Generalised dystonia	Generalised dystonia	Generalised dystonia	Decreased movement and dystonia,hyperactivity.	Decreased movement and extrapyramidal hypertonia,hyperactivity.	Focal hand dystonia which eventually became generalized dystonia	Dystonia, spastic hypertonia, clumsiness of movement and abnormal gait.	Dystonia,walk instability with a scissor gait and decreased exercise tolerance,twisting movements and abnormal postures in the body	Unstable muscle tone in the limbs, and hyperactive tendon reflexes.	19/19
Cerebellar ataxia	+	−	−	−	−	+	+	+	+	10/17
Dysarthria	+	+	+	−	−	+	+	−	−	8/14
Basal ganglia necrosis	Caudate and putamen	Caudate and putamen	Putamen	Caudate and putamen	Caudate and putamen	Putamen	Bilateral putamen	Putamen	Caudate and putamen	20/21
Subcortical white matter	−	−	−	NA	NA	NA	NA	−	−	
Spinal cord	−	−	−	NA	NA	NA	NA	NA	NA	
Cerebelar involvement	−	−	−	NA	NA	NA	NA	NA	NA	
MR spectroscopy lactate peak	+	NA	NA	NA	NA	NA	+	+	NA	
Biochemical investigation Lactate in plasma	1.63 mmol/ L	L 1.59 mmol/L	Not performed	2.3 mmol/L	2.5 mmol/L	NA	2.45 mmol/L	2.82 mmol/L	3.00 mmol/L	14/17
MRC CI (% of residual activity):	60% in muscle	Decreased enzimatic activity in muscle	Decreased enzimatic activity in muscle and in fibroblasts	NA	NA	NA	NA	NA	NA	11,12
Other symptoms	−	Peripheral demyelinating neuropathy	−	−	−	−	−	Knee hyperextension and foot eversion	Feet eversion, characterized by the outward deviation of the lesser toes	

P, patient; MRC CI, mitochondrial respiratory chain complex I; MRI, magnetic resonance image; NA, not available; N, no; P, patient; Y, years; m, months; LS, Leigh syndrome.

In this study, two pathogenic NDUFAF6 variants were identified in two Chinese patients with MC1DN17: c.362_364dupTGG (p. Val121dup) and c.169_190dupACTGACCACTACTGCCTGGAGC (p. Leu64fs*2). p. Val121dup was homozygous in patient 1 and compound-heterozygous with p. Leu64fs*2 in Patient 2. The c.169_190dupACTGACCACTACTGCCTGGAGC (p. Leu64fs*2) variant, located in exon 1, is absent from HGMD, HPSD, dbSNP, ExAC and gnomAD databases. It is predicted to introduce a premature termination codon, triggering nonsense-mediated mRNA decay (NMD), leading to significantly reduced mRNA and protein levels and resulting in a loss of function (LOF). This frameshift mechanism is functionally analogous to previously validated *NDUFAF6* null variants. Most notably, Sung et al. (29) systematically characterized *NDUFAF6* using deep mutational scanning and biochemical analyses, demonstrating that NDUFAF6 facilitates incorporation of NDUFS8 into complex I; their work provided experimental support of pathogenicity for seven novel *NDUFAF6* variants and functional evidence for over 5,000 additional variants, establishing that LOF variants consistently fail to restore ATP levels and complex I assembly (29). Similarly, Baidez-Maleyran et al. (24) showed that compound heterozygous NDUFAF6 mutations with reduced protein expression lead to dramatically reduced complex I enzymatic activity (40% reduction in mitochondrial ATP levels) and impaired mature complex I assembly, as assessed by blue-native PAGE (BN-PAGE) and in-gel activity staining. The p. Leu64fs*2 variant, by causing NMD and effectively abolishing protein production, is predicted to result in comparable functional consequences. The second variant, c.362_364dupTGG (p. Val121dup), is present in the Genome Aggregation Database (gnomAD v4.1.0) with a minor allele frequency of 0.000001859. Protein structure modelling indicates that p. Val121 resides within a critical multi-helix segment, where its duplication is predicted to directly disrupt local conformation, thereby severely impairing NDUFAF6 protein function. The pathogenic mechanism aligns with established functional data: Kohda et al. (17) demonstrated that transfection of wild-type NDUFAF6 into patient fibroblasts with missense variants successfully rescued complex I deficiency, confirming that structural alterations in critical domains disrupt function. Furthermore, Zhou et al. (28) utilized patient-derived immortalized B lymphocytes to confirm that structural variants affecting NDUFAF6 result in deficiency of mature supercomplex I, decreased cellular ATP production, and increased mitochondrial ROS. While the specific p. Val121dup variant is novel, deep mutational scanning by Sung et al. (29) established that variants affecting conserved structural residues consistently impair NDUFS8 incorporation and complex I biogenesis. The location of Val121 within a multi-helix segment critical for protein–protein interactions suggests that its duplication would similarly disrupt NDUFAF6’s scaffolding function during complex I assembly. In accordance with ACMG/AMP guidelines, the c.362_364dupTGG (p. Val121dup) variant is classified as likely pathogenic, and c.169_190dupACTGACCACTACTGCCTGGAGC (p. Leu64fs*2) is classified as pathogenic (Table 1).

The clinical features and genetic information of 24 patients with MC1DN17 are summarised in Table 2. A total of 18 different variants were identified, including 10 missense variants,1 nonsense variant, 2 splice site variants, 4 frameshift variants, and 2 small in-frame duplications. Approximately half of these patients carried the missense variant c.371 T > C(p. I124T). These variants disrupt the assembly of complex I and reduce its activity, leading to MC1DN17 (13, 14, 17, 18). Patients carrying these variants exhibit a highly homogeneous set of clinical symptoms. All individuals exhibited pronounced neurodevelopmental regression during infancy or early childhood (7 months to 6 years), characterized by the loss of previously acquired motor milestones, dystonia, and significant delay or loss of language. The two patients described in this report also demonstrated covert neurodevelopmental regression around one year of age. All patients (21/21) exhibited basal ganglia neuronal degeneration on MRI, and the imaging findings met the criteria for “bilateral striatal necrosis” (BSN) (30). Biochemically, 14 of 17 patients (including our patient) exhibited elevated blood lactate. Residual respiratory-chain complex (RCC) activity was reduced both in cultured fibroblasts (n = 9; range 40–86%) and in muscle (n = 6; range 28–80%). Compared to other etiologies, LS-associated BSN is often accompanied by brainstem involvement and an MRS lactate peak, which are highly suggestive findings (30). Among the available clinical data from these patients, all four cases exhibited a characteristic MRS lactate peak. Additionally, LS can involve the cerebellum, thalamus, spinal cord, and white matter, presenting with “BSN-plus” features such as stroke-like lesions, as well as cortical and cerebellar atrophy (5, 31, 32). Three patients also showed involvement of the cerebellar peduncles and dentate nuclei, three had parietal white-matter changes, and one had pontine lesions.

Mitochondrial DNA (mtDNA) variants are generally associated with high mortality (33). Importantly, patients carrying *NDUFAF6* variants have a better prognosis than those with variants in other nuclear DNA (nDNA) genes, showing lower mortality and longer preservation of motor function (10). All but one patient were alive at the time of publication; the exception died at 34 months (18). Notably, no clear genotype–phenotype correlation has been observed. Even carriers of different types of variants may present consistent clinical manifestations. The two patients in this study each carried the identical in-frame duplication variant c.362_364dupTGG (p. Val121dup), one in a homozygous state and the other in a compound heterozygous state. Patient 2 harboured an additional frameshift mutation predicted to cause more severe loss of function. Interestingly, both patients exhibited a similar age at onset and disease severity, whereas individuals sharing the same variant can differ markedly in these respects (18, 23). Moreover, the genotype does not adequately predict long-term outcome. The age of onset and disease progression in patients carrying these variants often demonstrate unpredictability. Given the current lack of reports on the long-term functional prognosis of NDUFAF6-related Leigh syndrome, accumulating more case data will help clarify its natural history, establish a reliable prognostic assessment system, and provide stronger evidence-based support for genetic counseling.

Regarding treatment and management, there is currently no curative therapy for Leigh syndrome (LS) caused by NDUFAF6 deficiency (24, 27, 28). Management remains primarily supportive and symptomatic, encompassing seizure control, management of dystonia, nutritional support, avoidance of metabolic stressors (e.g., infections), and individualized rehabilitation (27, 34). In select subtypes of mitochondrial disease, supplementation with thiamine (a cofactor for pyruvate dehydrogenase and *α*-ketoglutarate dehydrogenase), biotin (a cofactor for multiple carboxylases involved in energy metabolism), and coenzyme Q10 (a key electron carrier in the mitochondrial respiratory chain) has been attempted to enhance residual enzymatic activity or bypass metabolic blocks (35–37). Notably, biotin-thiamine-responsive basal ganglia disease (BTBGD) and primary CoQ10 deficiency caused by specific gene defects (e.g., *PDSS2*, *COQ2*, *COQ4*) have demonstrated biochemical and clinical responses to supplementation (36–39). However, no specific biochemical or symptomatic benefit from these supplements has been established in NDUFAF6-deficient LS to date (25, 27, 40). Consistent with this, Patient 2 in our cohort received supportive therapy including biotin, thiamine, and CoQ10, yet continued to experience progressive neurodevelopmental deterioration without observable clinical or metabolic improvement. These observations underscore the limited efficacy of current pharmacological interventions in this molecular subgroup and highlight the need for novel therapeutic strategies. At present, establishing a multidisciplinary team for long-term care aimed at improving quality of life and preventing complications remains the cornerstone of clinical practice for NDUFAF6-related LS.

This study has several limitations. First, long-term patient follow-up is required to assess disease progression. Second, the phenotypic spectrum of MC1DN17 remains incompletely defined due to the limited number of reported cases and a restricted variant spectrum; expanding the patient cohort should help clarify genotype–phenotype correlations and identify potential modifying factors. Third, although functional validation is important, this study did not include such experiments. Consequently, the genetic findings suggest an association with the observed phenotypes but do not yet establish causality. Further functional studies are therefore needed to elucidate the pathogenesis of MC1DN17. These studies should include validation of *NDUFAF6* variants, assessment of Complex I enzyme activity, blue-native polyacrylamide gel electrophoresis (BN-PAGE), or cellular respiration assays using patient-derived skin fibroblasts or muscle tissue.

In summary, this study reports on two Chinese patients with Leigh syndrome caused by two *NDUFAF6* variants, one of which is novel. This further extends the mutation spectrum of NDUFAF6-related Leigh syndrome. A comprehensive review of all reported cases indicates that this disorder has a highly consistent clinical profile, characterised by infantile or early childhood onset, subacute neurodevelopmental regression, dystonia and bilateral striatal necrosis visible on neuroimaging scans, as well as elevated lactate levels. Compared to Leigh syndrome of other etiologies, patients with NDUFAF6 deficiency exhibit lower mortality, longer preservation of motor function, and a relatively better prognosis. No clear genotype–phenotype correlations have been established, disease progression remains unpredictable, and there is currently no curative treatment, with management primarily consisting of supportive and symptomatic care. Future efforts should focus on collecting more long-term follow-up data to further elucidate the complete genotype–phenotype spectrum, natural history, and potential therapeutic targets.

## Data Availability

The raw data supporting the conclusions of this article will be made available by the authors, without undue reservation.

## Footnotes

1
https://swissmodel.expasy.org/

2
https://tgex.genecards.cn/
